# Evaluation of Endotracheal Tube Depth in the Out-of-Hospital Setting

**DOI:** 10.7759/cureus.13933

**Published:** 2021-03-16

**Authors:** Jeffrey S Lubin, Evan Fox, Scott Leroux

**Affiliations:** 1 Department of Emergency Medicine, Penn State Health Milton S. Hershey Medical Center, Hershey, USA; 2 Prehospital Care, Tower Health Reading Hospital, Reading, USA

**Keywords:** ems, endotracheal intubation, paramedic, airway management, prehospital

## Abstract

Introduction

Endobronchial intubation is a known complication of endotracheal intubation with significant associated morbidity and a reported incidence of up to 15%. In the out-of-hospital setting, paramedics must rely on bedside techniques to confirm appropriate endotracheal tube (ETT) depth. The present real-world practices of paramedics have not been described in this regard.

Methods

A multi-point survey was distributed to paramedics within the state of Pennsylvania. Participants were scored on the basis of their use of techniques to confirm ETT depth with the highest sensitivity to exclude endobronchial intubation.

Results

Four-hundred nine (409) responses from 111 emergency medical services (EMS) agencies were recorded. Participants were found to evaluate endotracheal tube depth via auscultation of bilateral breath sounds (91.7% of participants), visualization of the endotracheal tube as it advances 1-2 cm beyond the vocal cords (82.9%), observation of symmetrical chest rise (80.0%), and by securing the ETT at 21 and 23 cm at the incisors for women and men (18.6%). Experienced paramedics were more likely to use the 21/23 cm rule (p=0.039). Participants did not employ the cumulative use of these techniques (p < 0.001) as per a method that has been previously described to exclude endobronchial intubation with 100% sensitivity.

Conclusion

These data suggest that paramedics are not presently employing the most sensitive techniques to exclude endobronchial intubation. An educational initiative and protocol update may be beneficial.

## Introduction

The priority of endotracheal intubation in the prehospital setting is the establishment of a definitive airway, with appropriate positioning of an endotracheal tube (ETT) within the trachea to ensure adequate oxygenation and ventilation. In the prehospital setting, there is limited time to plan for the procedure and limited equipment to verify its success as compared to the hospital, where robust video laryngoscopy, chest radiography, and ultrasound is routinely available. The procedure is prone to improper positioning and the subsequent, potentially devastating, complications that follow [1].

Even if it can be assured that the ETT is positioned within the respiratory apparatus via end-tidal carbon dioxide monitoring and continuous pulse oximetry, this does not exclude endobronchial intubation as the ETT advances beyond the carina into the left or, more commonly, right mainstem bronchus. Endobronchial intubation is a potentially avoidable complication with a reported incidence of 2% to 15% [2-5]. If unrecognized, it may result in hypoxemia due to atelectasis of the contralateral lung, hyperinflation of the intubated lung, tension pneumothorax, and mortality [6-7]. The ideal placement of the ETT is a suggested 5±2 cm from the carina when the head is in the neutral position [8-9].

Paramedics must continue to rely on a physical exam and simple bedside techniques to ascertain ETT placement. It is well-established that positioning the ETT at the 21 cm and 23 cm marks at the upper incisors for men and women, respectively, reduces the incidence of endobronchial intubation, with reported rates of <1% to 2.4% [6,10]. Usual practices regarding the evaluation of proper ETT depth in the prehospital setting have not been extensively investigated. The purpose of the present study is to identify those techniques that are currently being practiced by paramedics within the state of Pennsylvania.

## Materials and methods

This study was reviewed and approved by our institution’s Institutional Review Board.

The research was conducted from December 30, 2019, through February 13, 2020, via an online survey that was hosted on a dynamic Web content service supported by our university. A list of certified paramedics throughout the commonwealth of Pennsylvania was generated by the regional emergency medical services (EMS) governmental authority, who then distributed a link to the survey via a one-time mass e-mail. Participation in the study was anonymous. Submission of the online survey constituted written consent for inclusion in the study.

Information was collected regarding the techniques each participant both practices and believes is mandated by local or state authority to evaluate ETT depth, the role of capnography in ETT depth verification, frequency of ETT reassessment, personal level of importance placed on ETT depth, and understanding of complications related to ETT depth. Demographic information was obtained regarding the number of years in practice, the number of intubations performed, and ground vs. air program participation.

For the purposes of determining the effect of intubation experience on implemented techniques, participants were demarcated as experienced if they performed a total of at least 20 intubations. This delineation was chosen on the basis of previous work, which describes a plateau in intubation success rate at approximately 20-25 intubations and an approximately 95% success rate at a cumulative experience of 20 intubations [11-13].

A scoring system was devised to rank participants’ cumulative use of appropriate techniques for determining ETT depth. This was derived, in part, from research data reported by Sitzwohl et al., who demonstrated sensitivities of 43%, 65%, 88%, and 100% to detect endobronchial intubation in the in-hospital setting using methods of observation of symmetrical chest rise, auscultation of bilateral breath sounds, tube insertion depth, and each modality in sum, respectively [14]. For the present scoring system, the following point values were assigned: No technique used: 0; visualizing the ETT as it is advanced 1-2 cm beyond the vocal cords: 1; observing symmetrical chest rise: 1; auscultating the chest - 2; “21/23” rule: 5. The sum was taken given the cumulative nature of the sensitivity described above.

The primary hypothesis of this study was that paramedics are not using the most sensitive technique to determine appropriate ETT tube depth. The method described by Sitzwohl et al. for 100% sensitivity and 95% specificity is to use auscultation, observation of symmetrical chest rise, and monitoring ETT insertion depth; this corresponds to a score of 8 in the present study. Statistical inference was performed using the R software package, version 3.6.1 (R Core Team, Vienna, Austria) [15]. The one-tailed Wilcoxon signed-rank test was used to test the hypothesis that the mean score among participants is less than eight.

A secondary hypothesis is that experienced paramedics are more likely to use the appropriate techniques for ETT depth verification than inexperienced. A Wilcoxon rank-sum test was used to compare mean scores between these groups. The Fisher exact test was used to report confidence intervals among the observed odds ratios.

## Results

Out of approximately 6,900 paramedics in Pennsylvania, 409 participants representing 111 EMS agencies responded during the study period. The mean years in practice among participants was 14.6, 11.0% were members of an air service, and 77.9% reported ≥20-lifetime intubations.

**Figure 1:**
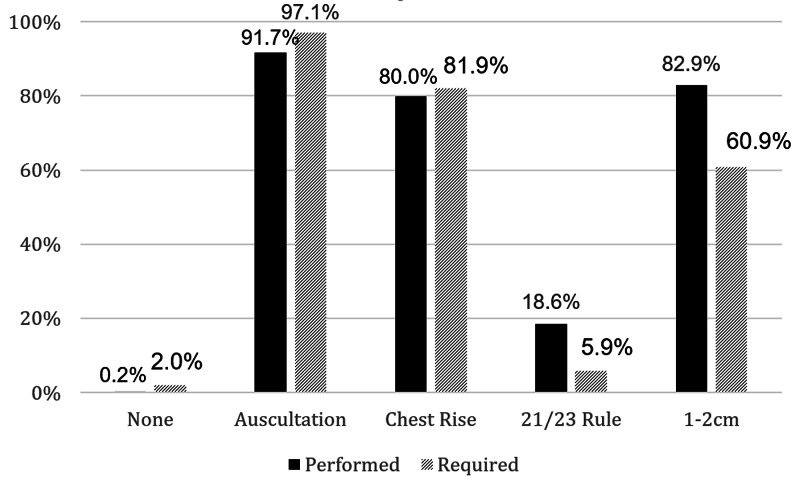
Techniques performed to determine appropriate endotracheal tube (ETT) depth vs. believed to be mandated by protocol, percentage of participants reporting Key: None = No techniques used; Auscultation = Auscultation of bilateral breath sounds; Chest rise = Observation of symmetric chest rise; 21/23 rule = ETT secured at 21 cm and 23 cm at incisors for women/men; 1-2 cm = Visualization of ETT cuff as it advances 1-2cm beyond the vocal cords

Each participant recorded the techniques currently in their practice along with their understanding of what is mandated for this by their appropriate governing body (Figure 1). The most common technique was auscultation (91.7%) while the least was the 21/23 cm rule (18.6%). Participants achieved a mean score of 4.39. This is less than a score of 8, which would correspond to the method with 100% sensitivity for endobronchial intubation as described by Sitzwohl et al. (p<0.001).

More participants (92.4%) reported having received education regarding ETT depth than those who had a recognition that ETT may still be malpositioned even if esophageal intubation is excluded (86.1%). Eighty-six point six percent (86.6%) reported the use of capnography in the course of their evaluation of ETT depth. Regarding the frequency of ETT reevaluation, 0.7% indicated that they never reassess ETT depth, 54.0% reassess only when the patient is moved or if there are signs of potential movement, and 45.2% reassess regularly (similarly to vital signs).

**Figure 2:**
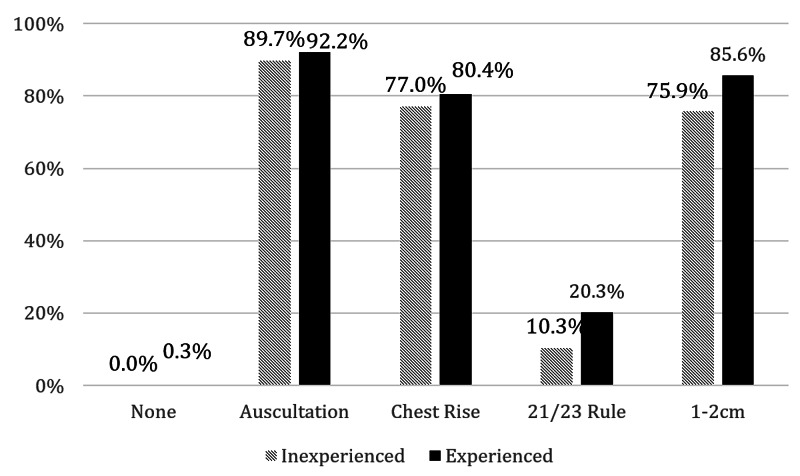
Techniques performed as grouped by inexperienced vs. experienced paramedics on a percentage basis Key: None = No techniques used; Auscultation = Auscultation of bilateral breath sounds; Chest rise = Observation of symmetric chest rise; 21/23 rule = endotracheal tube (ETT) secured at 21 cm and 23 cm at incisors for women/men; 1-2 cm = Visualization of ETT cuff as it advances 1-2 cm beyond vocal cords

A subgroup analysis was performed to compare experienced vs. inexperienced paramedics. Sixteen participants (3.9% of total) were excluded from this analysis on the basis of their reported “unknown” number of intubations. The mean years in practice were 3.2 in the inexperienced and 17.6 in the experienced groups. Figure 2 compares the practices for ETT depth evaluation between experience groups. Experienced paramedics overall achieved a higher evaluation score as compared to the inexperienced (4.52 vs. 3.84, p=0.005). They were, in particular, more likely to use the 21/23 cm rule for endotracheal tube placement (OR=2.20, 95% CI 1.02 to 5.27, p=0.04).

## Discussion

The present study suggests that paramedics who responded to the survey are not using the most sensitive techniques available in the out-of-hospital setting to evaluate ETT depth. Given that just 18.6% of respondents employ the 21/23 cm rule to evaluate ETT depth, the authors suggest an educational initiative or amendment to state protocol to include this rule.

Recent evidence suggests that intubation in the prehospital setting may be associated with increased morbidity and mortality, or at least no improvement in outcomes as compared to bag-valve-mask ventilation [16-17]. It may be the case that some complications of prehospital intubation might be obviated by increased training regarding ETT depth, although more research is needed.

Regarding the understanding of intubation protocol, just 9.8% of participants indicated a response consistent with what is required by Pennsylvania state: the sole mandate among the options provided in the survey is in auscultation of bilateral breath sounds [18]. However, there may be regional practice variations as per standard procedure or local medical direction. Moreover, most assumed a more conservative estimation of the protocol. Interestingly, more participants indicated that they have a requirement to use auscultation and observation of chest rise to evaluate depth (97.1% and 81.9%) than the percentage who endorsed routine practice of such evaluation modalities (91.7% and 90.0%, respectively).

It is reassuring that the overwhelming majority of paramedics indicate that they frequently reassess ETT depth. In sum, 99.3% make an effort to reassess with patient movement or on a regular basis, similar to vital signs. This is particularly important as an excursion of the ETT of approximately 1.9 cm caudad and cephalad, with flexion and extension of the neck, has been demonstrated [8].

There were several limitations to this study. Foremost, this is a self-guided, survey-based study and as such is subject to recall bias, response bias, and acquiescence bias; an observational study could add to the validity of the findings. Additionally, the survey was limited to distribution among paramedics in Pennsylvania. It is not clear if the results of this study are generalizable to other states, where practice and protocol variation exists. Finally, the scoring system described within to rank participants while an approximation of the likelihood to exclude endobronchial intubation as derived from results reported by Sitzwohl et al. has not yet been validated in the clinical setting.

## Conclusions

This study suggests that paramedics in Pennsylvania are not using the most sensitive techniques available in the out-of-hospital setting to evaluate endotracheal tube depth. There may be a need for improved education regarding endotracheal tube depth evaluation in the prehospital setting.
